# Coronary Occlusion Presenting One Week Post Bioprosthetic Surgical Aortic Valve Replacement and Diagnosed With OCT

**DOI:** 10.1016/j.jscai.2025.103602

**Published:** 2025-04-15

**Authors:** Bethany Wilkes, Ali Hillani, Angshuman Maulik, Tony Haddad, Wael Sumaya

**Affiliations:** a Department of Medicine, Dalhousie University, Halifax, Nova Scotia, Canada; b QEII Health Sciences Centre, Halifax, Nova Scotia, Canada

**Keywords:** Surgical aortic valve repair, complication, coronary artery occlusion, optical coherence tomography

## Clinical presentation

**Figure 1:**
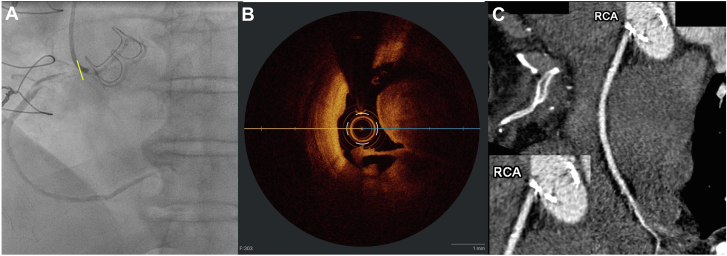
**Images from elective aortic valve replacement postoperative day 7.** (**A**) Angiogram of the subselected RCA showing an ostial filling defect, concerning for thrombus. The ostium appears to be behind the frame of the bioprosthetic aortic valve. The RCA was unable to be selected despite multiple attempts with different catheters. The yellow line indicates the approximate location of the OCT image. (**B**) OCT of the proximal RCA after balloon angioplasty, demonstrating a long length of thrombus. (**C**) Computed tomography coronary angiogram reconstruction of the RCA. The RCA appears to arise at or below the level of the valve sewing ring with at least part of the vessel covered, and contrast opacification of the coronary appearing to arise from the left ventricular outflow tract. Low-attenuation material at the ostium of the coronary may be related to thrombus or surgical material. Inset: magnification of the RCA ostium. OCT, optical coherence tomography; RCA, right coronary artery.

A 64-year-old man presented with sudden-onset chest pain, diaphoresis, dynamic troponin elevation, and an electrocardiogram showing atrial fibrillation and inferior ST-elevations. He was 7 days post elective aortic valve replacement (AVR; 25-mm Magna Ease bioprosthesis [Edwards Lifesciences]; discharged 2 days prior) for severe bicuspid aortic valve stenosis. Presurgical coronary angiography had shown minor nonobstructive coronary artery disease. He underwent urgent coronary angiography. The left system looked unchanged. However, it was not possible to select the right coronary artery (RCA), and subselective images demonstrated a new filling defect in the ostium of the RCA, concerning for thrombus (Figure 1A). A 3DRC guiding catheter was used. Attempts at thrombectomy were unsuccessful, leading to balloon angioplasty with a 2.5-mm balloon, which yielded improved flow but suboptimal results due to distal balloon slippage during inflation at the ostium. There was concern for potential external compression from the valve apparatus, so optical coherence tomography (OCT) was performed to further characterize the lesion, which revealed a long length of thrombus in the proximal RCA (Figure 1B, Supplemental Video 1). Further ballooning with a noncompliant balloon resulted in Thrombolysis in Myocardial Infarction (TIMI) grade 3 flow. Due to the ambiguous etiology, additional investigations were undertaken prior to stenting.

Computed tomography imaging revealed the RCA originated low in the right cusp, at or below the valve sewing ring, covering at least part of the ostium (Figure 1C, Supplemental Videos 2 and 3). Contrast suggested flow from the left ventricular outflow tract, while low attenuation at the RCA ostium possibly indicated the presence of thrombus or surgical material. The patient was started on systemic anticoagulation with plans for later RCA bypass, as he had stabilized and was pain-free. However, 6 days after admission, he experienced recurrent chest pain and inferior ST elevations, prompting immediate surgical intervention. One saphenous vein graft was placed at the proximal posterior descending artery. Postoperatively, the patient remained stable and had an uncomplicated recovery.

## Discussion

Ostial coronary occlusion is an uncommon but potentially life-threatening complication following AVR. Myocardial infarction (MI) within the first year of low-risk AVR has recently been reported at a rate of ∼2%,^1^^,^^2^ with a higher incidence of occlusions in the left coronary system.^3^ Presentations vary, including angina, ST-elevation MI, cardiogenic shock, and sudden cardiac death.^4^ Ostial stenosis most commonly manifests within 6 months after surgery,^3^ although delayed cases have been reported.^5^ Various mechanisms may contribute, including direct encroachment from the valve or associated materials, thromboembolism, mechanical injury, turbulent flow, immune response causing fibrosis, or septic emboli due to infective endocarditis.^3^^,^^6^^,^^7^ Additionally, mechanical injury from misplaced sutures or trauma during surgery may cause dissection or direct narrowing of the ostia. Determining the pathophysiology is key to selecting the most appropriate therapy. Intracoronary imaging such as OCT can be useful in differentiating external compression from intravascular causes of stenosis, such as plaque rupture. In this case, while an embolic phenomena secondary to atrial fibrillation is possible, the presence of proximal thrombus and absence of a left atrial thrombus on imaging render this etiology less likely.

Here, we believe the primary cause of ostial coronary stenosis was direct suture encroachment and the valve frame causing obstruction, restricting blood flow, and resulting in thrombus formation. This was supported by both OCT, which revealed a long thrombus within the lumen, and the computed tomography scan, which demonstrated the valve frame to be covering the coronary ostium with possible suture material near the ostium.

This case highlights the importance of multimodality imaging in determining etiology for early MI post AVR.

## Supplementary material

Supplemental Video 1 Optical coherence tomography imaging of the ostial and proximal right coronary artery showing thrombus. Blood artifact is present due to inability to fully engage the ostium.

Supplemental Video 2 Computed tomography coronary angiogram reconstruction of the right coronary artery demonstrating a focal wall irregularity along the inner anterior wall of the ascending thoracic aorta. The right coronary artery ostium demonstrated low attenuation. These findings were consistent with a possible cannulation site injury with small thrombus rather than spontaneous injury.

Supplemental Video 3 Computed tomography coronary angiogram, cross-sectional view demonstrating the right coronary artery ostium and bioprosthetic valve interface.
